# A precise navigation device for fixation of patella fractures with modified K-wire tension band:a comparative retrospective study

**DOI:** 10.1186/s13018-021-02235-6

**Published:** 2021-02-01

**Authors:** Fuming Wang, Haolan Xiong, Xiaotao Long, Yang Li, Xiaohua Chen, Gang Wang

**Affiliations:** 1 Department of Orthopaedics and Traumatology, Nanfang Hospital, Southern Medical University, Guangzhou, Guangdong People’s Republic of China; 2 Department of Orthopaedics and Traumatology, Chongqing General Hospital, University of Chinese Academy of Sciences, Chongqing, People’s Republic of China; 3 Department of anesthesiology, Chongqing General Hospital, University of Chinese Academy of Sciences, Chongqing, People’s Republic of China

**Keywords:** Navigation device, Patella fractures, Modified K-wire tension band

## Abstract

**Background:**

Traditionally, the technique of modified tension band wires (MTBW) has been the most commonly used surgical procedure. The purpose of this study is to design a precise navigation device that can obtain a standard position of K-wires for (MTBW) and to compare the precise MTBW (P-MTBW) by a navigation device with the conventional MTBW (C-MTBW) by hands in a retrospective study.

**Methods:**

The device was designed by solidworks2012 software (USA), which could provide a precise guidance for obtaining parallel K-wires. Besides, it could set the distance between two K-wires and the level of K-wires below patellar anterior surface. From June 2014 to August 2018, a total of 112 patients were employed in this retrospective study. The patients were divided into P-MTBW group and C-MTBW group according to the surgical technique with or without the precise navigation device. We needed to record and analyze the operation time and the number of fluoroscopy, postoperative internal fixation imaging, knee function and complications.

**Results:**

There were 54 patients in P-MTBW group and 58 patients in C-MTBW group. There were statistically significant differences (*P* < 0.001) in the operation time between P-MTBW group (39.5 ± 4.7; range, 32–49 min) and C-MTBW group (53.7 ± 6.8; range, 42–71 min). The number of intraoperative fluoroscopy was significantly less (*P* < 0.001) in P-MTBW group (4.2 ± 1.4) versus that of C-MTBW group (8.3 ± 2.7). According to Iowa knee score, there was no significant difference (*P* = 0.268 at 1 year) in function between the two groups. According to our own evaluation criteria for MTBW, anyone in the P-MTBW group was excellent and 26 patients were excellent, 20 patients were good, and 2 patients were fair in the C-MTBW group.

**Conclusion:**

The navigation device can reduce operation time and intraoperative fluoroscopy frequency. P-MTBW fixation is an accurate and effective surgical procedure for patella fractures.

## Introduction

Patella fractures account for approximately 1% of fractures on adults [1, 2]. The transverse fracture is the most common type of patella fracture, which often causes functional disability of the knee extensor apparatus with the displacement of the fractured fragments [3].

Traditionally, the technique of modified tension band wires (MTBW) has been the most commonly used surgical procedure and is considered the gold standard for transverse patella fracture [4–7]. This technique consists of two parallel K-wires perpendicular to the fracture line and an eight-shaped wire passing anteriorly over the patella and behind the K-wires. According to the AO principle [8, 9], only when the two K-wires remain parallel can convert the tension force of the anterior patella into a compressive force across the articular surface, thus promoting fracture healing. Although the classic MTBW technique is reserved for transverse fractures, certain comminuted fractures can also be treated with a tension band construct if the articular surface is intact enough to allow for compression [10].

Parallel K-wires are the key to the tension band technique, usually depending on the experience and hand feeling of the surgeon. There are no reports about a device that remains two K-wires absolutely parallel; hence, we have designed a precise device to simplify surgery. This device can provide a precise guidance to obtain a parallel K-wires placement in any configuration, which can set the distance between two K-wires and the level of K-wires below patellar anterior surface, thus reducing the operation time and intraoperative fluoroscopy. The goal of this study is to compare the precisely modified tension band wiring (P-MTBW) by a navigation device with the conventionally modified tension band wiring (C-MTBW) by hands in a retrospective study. We hypothesize that the P-MTBW would exhibit a better performance regarding surgical time, intraoperative fluoroscopy, functional score and fewer complications than that of C-MTBW.

## Materials and methods

### Design of the guide device (Fig. 1)

The device was designed by solidworks2012 software (USA). The design process turned to professional engineers, which could meet the functional requirements of the design and fit for industrial production. Two U-shaped arms of the device constitute the main structure through the cross bar c. Sleeves a and b are both divided into inner and outer doubled layer sleeves, while the outer sleeve is fixed on the main structure of the device and the inner sleeve can slide onto the outer sleeve. The centerline of Two U-shaped arms is controlled by the main structure of the device. The plane of the bottom of the tray e is parallel to the plane of the centerline of sleeves a and b, and both bar c and rod d have rulers. The tray e touches the front surface of the patella, and the distance between the plane of K-wires and the front surface of the patella is adjusted by sliding rod d. The distance between sleeves a and b is adjusted by the cross bar c.

**Fig. 1:**
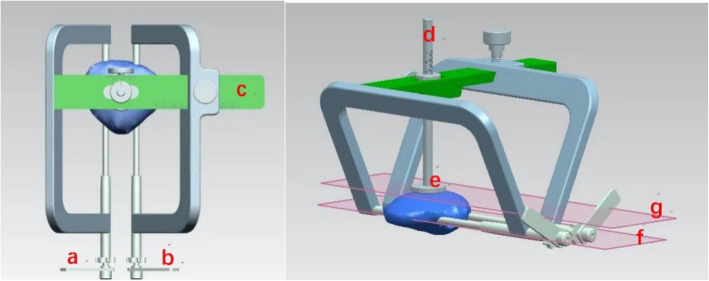
Design appearance of the navigation device

### Clinical study

This study protocol was approved by our hospital Ethics Committee. This retrospective study reviewed patients with patellar fractures who underwent MTBW surgical treatment from June 2014 to August 2018. The inclusion criteria are shown as follows: (1) transverse fractures with or without a single additional fragment; (2) 18 to 65 years without previous knee surgery; (3) the articular displacement is greater than 2 mm or fragment separation is greater than 3 mm on radiography; (4) C1 and C2 type with consideration to AO classification; (5) MTBW with or without a navigation device; (6) follow-up at least 12 months. The exclusion criteria: (1) associated with a fracture of the distal femur or tibial plateau; (2) previous knee diseases such as osteoarthritis. According to these criteria, 112 patients were included in the study. The patients were divided into C-MTBW and P-MTBW according to the surgical technique with or without the precise navigation device. There were 58 patients treated with the C-MTBW method and 54 patients treated with P-MTBW by the precise navigation device. We needed to record and analyze the operation time and the number of fluoroscopy, postoperative internal fixation imaging, function, and complications.

**Fig. 2:**
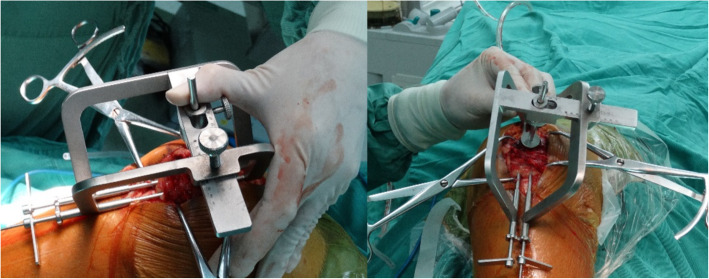
The physical appearance of the navigation device during the operation

All patients underwent the standard MTBW technique. Approach and reduction techniques depend on the standard technique according to the AO principle, while the difference was that the P-MTBW group uses a self-designed precise navigation device to guide the K-wires. Adjusting the device parameters according to the width and thickness of patella measured before surgery. The distance of K-wires was set at one third of the widest diameter of the patella, and the level of the K-wires was set according to the thickness of the outer third of the patella. After setting the parameters of device, K-wire was implanted through the sleeve (Fig. 2).

### Postoperative management and evaluation

For all patients, an elastic bandage was used for 48 h after surgery for reducing swelling. Isometric quadriceps exercise and straight leg raises were started when pain had subsided. The knee joint was protected by the knee adjustable brace that allows 60° of motion after surgery, 90°of motion after 2 weeks and no restriction after 4 weeks. Postoperative follow-ups were arranged for one and 2 weeks, 1, 2, 3, 6, and 12 months, and the amount duration was longer than 1 year. Common complications included incision infection, failure of internal fixation, fracture displacement, and K-wire irritation. After 1 year of surgery, the knee function was evaluated according to the Iowa knee score criteria.

Postoperative imaging usually was used to evaluate the reduction of the fracture, which ignored the assessment of the internal fixation position. We believe it is necessary to establish a standard evaluation strategy for K-wires of MTBW technique. All patients should have standard X-rays of the knee on two planes after surgery. An AP viewing and a lateral view are evaluated according to the angle between the K-wires. The angle is measured by two senior doctors respectively. If the error is within 2°, the average value is taken. If the error exceeds 2°, we seek help from the third doctor. If the angle at both views is less than 5°, it is defined as excellent. If the angle at one view is between 5° and 15 °, it is defined as good. If the angle at both views is between 5° and 15 °, it is defined as fair. If the angle at any view is more than 15 °, it is bad.

Data were presented as mean ± SD for continuous variables and the number of categorical variables. To compare two groups, there was a two-sample *t* test used for continuous variables, Pearson chi-square tested for categorical variables, and Wilcoxon rank sum tested for ordinal categorical variables. The differences were presented as mean (95% CI) for continuous variables and odds ratio (95% CI) for categorical variables. The significance level was set at 0.05 for all the tests, and data was entered and analyzed with SPSS21.0 statistical software (SPSS Inc., Chicago, IL, USA).

## Result

There were 54 patients in P-MTBW group and 58 patients in C-MTBW group, 3 cases in P-MTBW group and 5 cases in C-MTBW group lost to follow-up. Demographics of both groups were no statistically significant differences (Table 1). There were statistically significant differences (*P* < 0.001) in the operation time between P-MTBW group (39.5 ± 4.7; range, 32–49 min) and C-MTBW group (53.7 ± 6.8; range, 42–71 min). The number of intraoperative fluoroscopy was significantly less (*P* < 0.001) in the P-MTBW group (4.2 ± 1.4) versus that of C-MTBW group (8.3 ± 2.7). According to Iowa knee score, there was no significant difference (*P* = 0.752 at 1 month, *P* = 0.836 at 3 months, *P* = 0.363 at 6 months, *P* = 0.268 at 1 year) in the function between the two groups (Table 2).

**Table 1:** Clinical demographic data between the 2 groups

Variables	P-MTBW(54)	C-MTBW(58)	Test statistics	*P* value
Age (years)	38.5 ± 12.7	40.5 ± 12.0	− 1.05	0.297
Gender				
Male	35	33	0.735	0.391
Female	19	25		
Operation time	39.5 ± 4.7	53.7 ± 6.8	− 11.3	*P* < 0.01
No. of fluoroscopy	4.2 ± 1.4	8.3 ± 2.7	− 10.1	*P* < 0.01
Complications	9	11	0.101	0.751

**Table 2:** According to Iowa knee score

Follow-up time		1 month		3 months		6 months		1 year	
Function									
		PMTBW	CMTBW	PMTBW	CMTBW	PMTBW	CMTBW	PMTBW	CMTBW
**Iowa** **knee** **score** **criteria**	90–100	5	4	27	28	35	33	38	35
	80–89	22	25	14	15	12	14	11	12
	70–79	19	18	7	5	3	4	2	5
	≤ 69	8	11	4	7	1	3	0	1
Test statistics		− 0.316		− 0.207		− 0.911		− 1.107	
*P* value		0.752		0.836		0.362		0.268	

According to our own evaluation criteria, all patients in the P-MTBW group were excellent, and 26 patients were excellent, 20 patients were good, and 2 were fair in the C-MTBW group. The comparison between the two groups was statistically significant (*P* < 0.001) (Table 3).

**Table 3:** Evaluation of the accuracy of the guide device

Variables		P-MTBW	C-MTBW	Test statistics	*P* value
K-wires of MTBW evaluation strategy	< 5°in both views	54	26	− 6.408	*P* < 0.001
	5–15°in one view	0	30		
	5–15°in both views	0	2		
	> 15°in any view	0	0		

There was no significant difference in the incidence of complications(*P* = 0.751)between the two groups, and none of all patients in this study were infected. One patient experienced failure of internal fixation due to steel wire breakage, five patients had K-wire irritation, and three were presented with fracture displacement in the P-MTBW group. In the C-MTBW group, three patients had internal fixation failure due to wire breakage and K-wire withdrawal, six patients had K-wire irritation, and two presented with fracture displacement. The removal rate of internal fixation was not calculated in this study because of custom.

## Discussion

Although there are new techniques such as cannulated screw tension band [11, 12],

cable pin system [13], and patella plate [14], the MTBW is still the most widely accepted surgical method for the treatment of patellar transverse fracture [7]. The MTBW technique is easy to operate on and economical to reduce the burden on patients, and the key of MTBW technique is to keep two K-wires parallel [8, 15]. The manual operation is not enough to keep two K-wires parallel and needs to be repeatedly adjusted; hence, we design and create this precise guiding device. Besides, it can set the distance between two K-wires and the level of K-wires below patellar anterior surface. According to the anatomic parameters of preoperative measurement, it can set distance accurately in the navigation device. Our study shows that the navigation device is accurate and effective, which can reduce the operation time and intraoperative fluoroscopy frequency and is beneficial to the treatment of patellar transverse fracture.

So far, there is no research on evaluating the quality of MTBW technique; hence, it is necessary to establish an evaluation strategy for K-wires of MTBW technique. The MTBW technique should be evaluated according to the angle between the two K-wires in an AP view and a lateral view after surgery. There is only 45% (26/58) excellence in C-MTBW group and all are excellent in P-MTBW group. The level of the K-wires and the distance between two K-wires are not included in the evaluation strategy, because a complex evaluation strategy is not easy to accept and understand. The evaluation strategy for MTBW technique is highly necessary, so that we can adjust the position of K-wires during the operation.

According to the AO principle [8], the ideal level for the K-wires lies in the center of the patella, approximately 5 mm below its anterior surface. In practice, the K-wires are closer to the articular than to the anterior surface. Hsu [16] reported the depth of K-wires involving 170 patients treated with MTBW: 37 (22%) patients at superficial third and 133 (78%) patients at the middle third of the patella and superficially placed Kirschner wires increased the rate of minor loss of reduction.

There is a recently published study by Yang [15] et al. show that the wide distance between them and the K-wires at the deep level might be more helpful for MTBW. Ling [17] performed a finite element analysis, and posteriorly placed K-wires make optimal stability possible. We believe that it should be located 5 mm below the anterior surface of the lateral third, because the anterior surface of the patella is irregular and the lateral part of the patella is thinner.

There are some limitations to our study. First, it is not a prospective and randomized study. Second, all the operations were open, and the closed reduction and internal fixation guided by the guidance should be further carried out in the later. Finally, we do not distinguish the level of K-wires and the distance between the K-wires; finite element analysis [18, 19] can be carried out for comparison in the future.

In conclusion, the navigation device can reduce the operation time and intraoperative fluoroscopy frequency. P-MTBW fixation is an accurate and effective surgical procedure for treatment of transverse patellar fractures.

## Data Availability

All of the data are available in contact with the correspondent author.

## Abbreviations

K-wire: Kirschner wire
MTBW: Modified tension band wires
P-MTBW: The precise MTBW by a navigation device
C-MTBW: The conventional MTBW by hands
